# Preparing for and responding to sexual and reproductive health in disaster settings: evidence from Fiji and Tonga

**DOI:** 10.1186/s12978-021-01236-2

**Published:** 2021-09-20

**Authors:** Kristen Beek, Robyn Drysdale, Matthew Kusen, Angela Dawson

**Affiliations:** 1 School of Population Health, Faculty of Medicine and Health, University of New South Wales, Sydney, Australia; 2 Humanitarian Programme, International Planned Parenthood Federation (IPPF), Suva, Fiji; 3 Bangkok, Thailand; 4 Centre for Australian Public and Population Health Research, Faculty of Health, University of Technology Sydney, Level 8, Room 225, 235 Jones St, PO Box 123, Sydney, NSW 2007 Australia

**Keywords:** Sexual and reproductive health, Pacific Islands, Humanitarian crisis, Preparedness, Capacity building, Disaster response

## Abstract

**Background:**

Pacific Island countries are vulnerable to disasters, including cyclones and earthquakes. Disaster preparedness is key to a well-coordinated response to preventing sexual violence and assisting survivors, reducing the transmission of HIV and other STIs, and preventing excess maternal and neonatal mortality and morbidity. This study aimed to identify the capacity development activities undertaken as part of the SPRINT program in Fiji and Tonga and how these enabled the sexual and reproductive health (SRH) response to Tropical Cyclones Winston and Gita.

**Methods:**

This descriptive qualitative study was informed by a framework designed to assess public health emergency response capacity across various levels (systems, organisational, and individual) and two phases of the disaster management cycle (preparedness and response). Eight key informants were recruited purposively to include diverse individuals from relevant organisations and interviewed by telephone, Zoom, Skype and email. Template analysis was used to examine the data.

**Findings:**

Differences in the country contexts were highlighted. The existing program of training in Tonga, investment from the International Planned Parenthood Federation (IPPF) Humanitarian Hub, the status of the Tonga Family Health Association as the key player in the delivery of SRH, together with its long experience of delivering contract work in short time-frames and strong relationship with the Ministry of Health (MoH) facilitated a relatively smooth and rapid response. In contrast, there had been limited capacity development work in Fiji prior to Winston, requiring training to be rapidly delivered during the immediate response to the cyclone with the support of surge staff from IPPF. In Fiji, the response was initially hampered by a lack of clarity concerning stakeholder roles and coordination, but linkages were quickly built to enable a response. Participants highlighted the importance of personal relationships, individuals’ and organisations' motivation to respond, and strong rapport with the community to deliver SRH.

**Discussion:**

This study highlights the need for comprehensive activities at multiple levels within a country and across the Pacific region to build capacity for a SRH response. While the SPRINT initiative has been implemented across several regions to improve organisational and national capacity preparedness, training for communities can be strengthened. This research outlines the importance of formalising partnerships and regular meetings and training to ensure the currency of coordination efforts in readiness for activation. However, work is needed to further institutionalise SRH in emergencies in national policy and accountability mechanisms.

## Plain Language Summary

Pacific Island countries experience many disasters, including cyclones and earthquakes. The International Planned Parenthood Federation (IPPF) has been working in the Pacific to help build skills to improve the response to sexual and reproductive health (SRH) and rights during disasters. This paper describes research to identify capacity building activities that helped prepare organisations in Fiji and Tonga and how this affected the delivery of SRH during Tropical Cyclone Winston in 2016, and Tropical Cyclone Gita in 2018. Key informants in senior positions from relevant organisations were recruited and interviewed by telephone, Zoom, Skype and email. We used a framework that described different levels of capacity across phases of the disaster management cycle to analyse the data. Eight key informants described differences in Fiji and Tonga’s preparedness activities before Tropical Cyclones Winston and Gita that affected the way in which services were delivered. The Tonga Family Health Association was well established as a key player in SRH service delivery before Gita and had built relationships and delivered training for disaster response to staff across a number of organisations including the Ministry of Health (MoH). These preparedness efforts facilitated a smooth and rapid response. In Fiji, the response was initially affected by a lack of training, role clarity and coordination, but linkages were quickly built to deliver care and services. Participants highlighted the importance of personal relationships, individuals’ and organisations’ motivation to respond, and strong links with the community to deliver SRH care. This study highlights the need for inclusive activities at individual, organisational and national levels within countries and across the Pacific region to build capacity for a SRH response.

## Background

Pacific Island countries (PICs) and territories are some of the most vulnerable to natural hazards, the effects of which are exacerbated by poor development and climate change [1]. Many PICs are situated within or close to the Typhoon Belt and the boundary between the Australian and the Pacific tectonic plates, increasing the risk of cyclones, hurricanes, flooding, earthquakes, tsunamis, and volcanic eruptions [2]. The sexual and reproductive health and rights (SRHR) of women, girls, men, and boys and gender diverse individuals are significant health concerns in all humanitarian contexts, including those caused by natural hazards. The risk of sexual violence increases in insecure and unstable settings and in contexts where protection from legal, social and community support systems have been undermined by displacement or disruption [3]. Humanitarian contexts may increase risk factors for sexually transmitted infections (STIs), including HIV and disrupt access to treatment and prevention services [4]. Maternal mortality is reportedly ten to 30 percent higher in humanitarian contexts compared with non-crisis settings [5]. In these contexts, women and girls will often give birth without skilled birth assistance or necessary resources, increasing the risk of preventable mortality and morbidity. A lack of access to newborn care can also jeopardise infant survival [6].

**Fig. 1:**
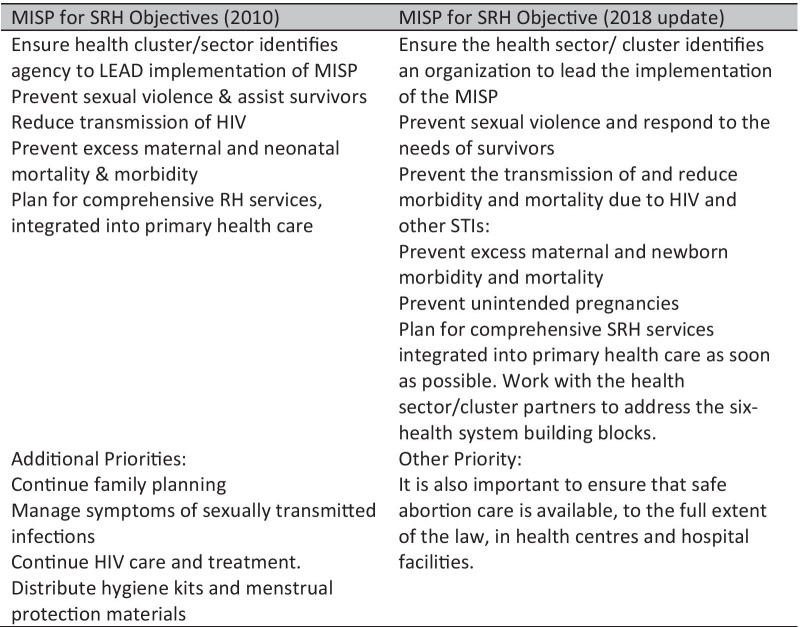
Objectives of the Minimum Initial Service Package for Sexual and Reproductive Health in Crisis

In response to these critical health needs, the Inter-agency Working Group on Reproductive Health in Crises (IAWG) has developed a set of objectives, activities, information, and resources focused on: preventing sexual violence and assisting survivors, reducing the transmission of HIV, and managing STIs, preventing excess maternal and neonatal mortality, preventing unintended pregnancies, and moving to comprehensive SRH services as soon as possible [7]. These aims are encompassed in the Minimum Initial Service Package for Sexual and Reproductive Health in Crisis Situations (MISP), a coordinated set of priority activities to be delivered in response to SRH needs. The Sphere Humanitarian Charter and Minimum Standards in Disaster Response have incorporated the MISP for SRH as a minimum standard of care in humanitarian response [8]. The MISP for SRH was initially proposed in the mid-1990s, and updated objectives and activities were included in the Inter-agency Field Manual on Reproductive Health in Humanitarian Settings of 2010 and most recently in 2018 (See Fig. 1).

In 2007, IPPF, with support from the Australian Government, launched the Sexual and Reproductive Health in Crisis and Post-Crisis Situations (SPRINT) initiative. SPRINT was established to improve the health outcomes of crisis-affected populations, focusing on reducing SRH-related morbidity and mortality. This initiative is led by IPPF in collaboration with its Member Associations and other national and international partners and is dedicated to building country capacity to implement global standards, including the MISP, in crisis contexts [9]. IPPF is the world’s largest federated reproductive health Non-government organisation, providing SRH services in more than 160 countries with a strong presence in nine Pacific Island Countries [10]. The current phase of the SPRINT initiative is implemented with 13 locally-owned Member Associations, including the Reproductive and Family Health Association of Fiji (RFHAF) and the Tonga Family Health Association (TFHA) in the Pacific [11].

Since its launch, there have been three phases of SPRINT (2007–2021). Activities under the initiative have included advocacy for the MISP, MISP coordination, capacity building, institutional strengthening, and SRH in Emergency (SRHiE) service delivery/response. The program aims to raise the profile of the MISP and promote a comprehensive approach to reproductive health that considers pre, during, and post-crisis phases. The establishment and on-going support of national SRH coordination mechanisms and the provision of capacity building and tools to prepare and respond in the acute phases of crises are central to these aims. SPRINT also works to support the integration of the MISP into country emergency response and disaster risk reduction policies.

In 2017, IPPF established its Global Humanitarian Hub in Bangkok and the Pacific Humanitarian (sub-) Hub in Suva. These coordinate humanitarian work across the East and Southeast Asia and Oceania Region (ESEAOR) and the South Asia Region (SAR) in collaboration with Member Associations and other national and international partners. In 2018, IPPF launched its global Humanitarian Strategy (2018–2022), demonstrating a commitment to an integrated and comprehensive approach to SRHR in emergencies and linking this work to its long-term development mandate [12]. A critical component of this is the “colocation of the Sub-Regional Office for the Pacific (SROP) and Pacific Humanitarian Hub in Suva to coordinate and share lessons between humanitarian and development programming in the Pacific” [12]. The United Nations Population Fund (UNFPA) plays a critical role in supporting the work of IPPF at the regional and country-level by prepositioning reproductive health kits containing essential drugs, basic equipment and supplies needed to provide SRH care in crise [13].

The Pacific Island countries of Fiji and Tonga regularly experience cyclones, and in the period 2016–2018 had introduced preparedness measures under the SPRINT program. On the 20th of February 2016 Severe Tropical Cyclone Winston, the most intense tropical cyclone (category 5) in the Southern Hemisphere on record, reached maximum intensity near Fiji, causing extensive damage and 44 deaths. On the 12th of February 2018, Category 4 Tropical Cyclone Gita, the worst the country had experienced in 60 years, peaked, severely impacting Tonga. This paper reports on research that examined capacity development activities undertaken as part of the SPRINT program in Fiji and Tonga, and how these enabled the SRH response to Cyclone Winston and Cyclone Gita. This study identifies the different approaches to capacity building and response in the two settings and delivers recommendations for future efforts and investment in line with the objectives of the MISP.

## Methods

This descriptive qualitative study involving eight key informant interviews sought to identify activities that were carried out by SPRINT partners, including IPPF Member Associations (RFHAF and TFHA), other non-government organisation (NGO) and community-based organisations, and the Ministries of Health in Fiji and Tonga to foster workforce, organisational and community capacity development before Cyclone Winston (2016) and Cyclone Gita (2018). In addition, interview questions explored how these activities influenced the type, scope, and timeline of SRH response to these cyclones and mitigated challenges to delivering the MISP. We used the reporting guide outlined by O’Brien et al. [14] to present our findings.

**Fig. 2:**
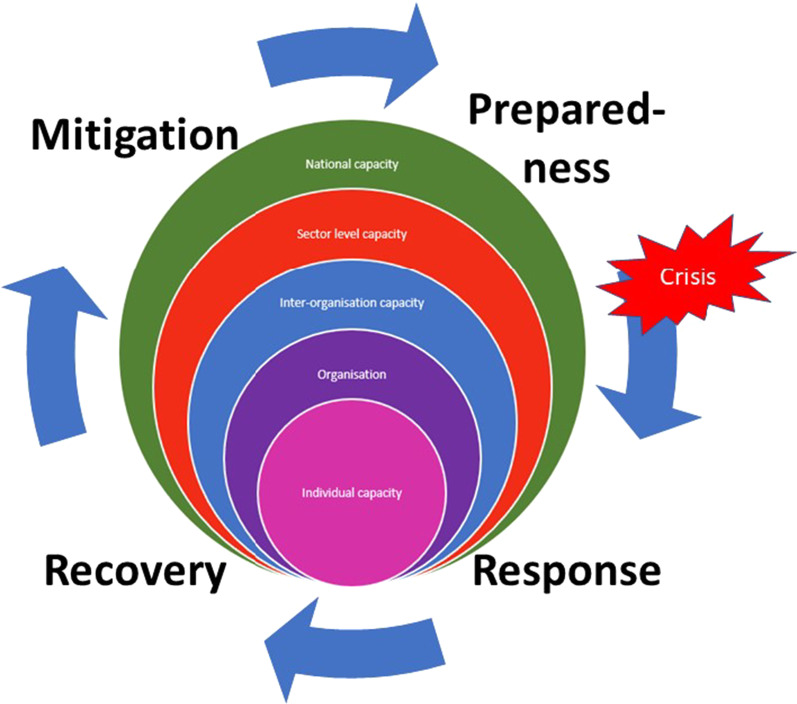
A framework of capacity building in SRH in emergencies adapted from [16]

In this paper, we define capacity development as efforts to improve the knowledge and skills of those providing SRH care, information, and services, building support and infrastructure for organizations, and developing partnerships with communities [15]. The research was informed by a framework designed to assess public health emergency response capacity [16] across various levels (systems, organizational, and individual) and the phases of the disaster management cycle (preparedness, response, recovery, mitigation) (see Figs. 1, 2). This study was, however, only concerned with the preparedness and response phases.

### Study setting

Fiji and Tonga were selected as case studies to explore preparedness and response to SRH needs in crises. Both countries have a shared experience of tropical cyclones but have different cultural and demographic contexts. Fiji is a Melanesian country with a population of 897,295 (across approximately 100 inhabited islands), while Tonga is a Polynesian country with a population of 105,845 (across 36 inhabited islands) [17, 18]. While both are upper-middle-income countries [19] and have youthful populations, Tonga is more densely populated (49/km^2^ compared with 147/km^2^ in Fiji). SRH indicators also differ across the nations. Fiji has a contraceptive prevalence rate (CPR) of 30 percent, while Tonga’s CPR is 17%. Adolescent fertility rates are similar in both Fiji and Tonga (49 vs. 30 births per 1000 women 15–19 years) [17], while the percentage of women subjected to physical and sexual interpersonal violence in their lifetime (2000–2015) differs (64% vs. 40%) respectively [20].

The Fiji National Disaster Management Office (NDMO) is the Fiji government's coordinating body for natural disasters. While the Ministry of Health and Medical Services has identified maternal, newborn and adolescent care and gender-based violence amongst the top health priorities in a reproductive health response [21], SRH in emergencies (SRHiE) is absent from the Fiji Ministry of Health Reproductive Health Policy [22] and there are SRH-related gaps in the Fiji National Disaster Management Plan [23] that was current at the time of Cyclone Winston. The emergency management and response structure in Tonga is led by the National Disaster Council (NDC) and directed by a national plan that does not include SRH [24]. Disaster management is noted in a generic manner in the National Health Strategic plan [25] while SRHiE is identified in a government SRHR needs assessment [26] published before Cyclone Gita. Both countries have adopted a National Cluster System based on the UN model. The key clusters involved in any SRHiE response include the Health and Nutrition/Health, Nutrition and Water, Sanitation and Hygiene cluster (led by the national Ministry of Health and co-led by WHO and UNICEF) and the Safety and Protection cluster (led by the national Ministry of Women and co-led by UN women). At the time of cyclone Winston and Gita, the 2010 version of the MISP for SRH (see Fig. 1) was the standard applied in both responses.

### Recruitment

Study informants were recruited purposively to engage individuals from key organisations, and included staff who were directly involved in the preparedness and response efforts to cyclones Gita and Winston. We sought a diversity of perspectives, including government and NGO workers, across both countries’ health and disaster response sectors. Information about the study was sent to key individuals with an invitation to participate in an interview. During the recruitment and data-gathering processes, several communication challenges were experienced due to the interviewers’ remoteness, which made it difficult to establish contact with respondents and develop rapport. These were overcome by multiple contacts and discussions with individuals over a 6 month period.

### Data gathering

The findings of a desk review informed the development of questions for the interviews and helped identify possible participants. A stakeholder reference group were invited to provide input into the interview questions, and these were piloted in January 2020. Due to the COVID-19 pandemic, Australia closed its international borders in March 2020, prohibiting travel to Tonga and Fiji. As a result, interview data were collected via telephone, Zoom, Skype, and email. Multiple contacts with key informants enabled thick descriptions to be built and saturation to be reached through concurrent analysis that identified no new patterns emerging. Rigor was also sought by inviting some informants to check the data for credibility. KB and AD met regularly to discuss the data and ensure a detailed audit trail was collected. Due to the small number of informants and unique context, respondents have been de-identified as much as possible to ensure confidentiality. To maintain anonymity, direct quotes included in this report are not attributed to individuals.

### Analysis

Data were analysed using a template as described by King [27]. Coding was directed according to categories that aligned with the aims of the study and the process was managed using the qualitative research software QSR Nvivo 12. An initial template was developed based on the list of codes to identify themes in the textual data and these were modified as the analysis continued. The Framework of Factors Influencing SRHiE Response, together with a broad understanding of capacity and capacity development programming (see Fig. 1) informed the template used for data analysis. This allowed for the consideration of a wide range of factors that may influence the effectiveness of SPRINT-supported training, other capacity development efforts, and the response.

### Ethical approval

This study was granted ethical approval by the Human Research Ethics Committee of the University of Technology Sydney, Fiji National Health Research Ethics Committee, and the Tonga National Health Ethics and Research Committee.

## Findings

Eight key informants were interviewed for this study. We outline the findings according to the preparedness and response phases.

### Preparedness: before cyclones Winston and Gita

#### Fiji

Before Tropical Cyclone Winston in Fiji, key informants reported that few capacity development activities had been implemented to support the delivery of the MISP. Staff from the IPPF Sub-Regional Office for the Pacific (SROP), the Member Association RFHAF and partners were involved in the response to Winston and of these, only one responder had received training on the MISP. This training had been provided during the second phase of the SPRINT Initiative and a significant amount of time had passed since the completion of this training, with no follow-up refresher training or opportunity for the individual to apply their new knowledge or skills. Staff from the SROP were familiar with the MISP due to their involvement in reporting and supporting regional humanitarian work. However, at that time they had not received a formal orientation to the package.

At the onset of the cyclone, two surge capacity staff members from IPPF were deployed to Fiji to conduct a ‘crash course’ for responders on the basics of the MISP and coordination skills needed to support the response in Fiji. These staff members had been involved in implementing SRH services during crises in different contexts. A key informant stated:

…the crash course in Fiji it was really focused on coordination. And how to handle yourself and your staff in crisis situations. How to be more tolerant, more strategic, and how to react quickly, to how fast things are the way things change. So it was really preparing them psychologically and emotionally on what would happen. Because family planning, HIV, maternal health, SGBV, they’ve been doing this for how many years… They know this stuff.

Participants appreciated the practical nature of this training, with one explaining that “when we did the crash course, they focused on what we would be doing” (Respondent). Further capacity development strategies were deployed to ensure those involved in the response, including nursing staff and volunteers were familiar with where their tasks fit within the MISP implementation, to clarify roles, and to explain each medical mission's processes and procedures. In addition to these formal training sessions, these two experienced IPPF staff-members remained with the in-country and SROP-supported response teams for ten days to advise, guide, debrief and build daily on lessons learnt.

#### Tonga

In Tonga, key informants reported that training had been implemented well before the onset of cyclone Gita. This training had been conducted alongside other preparedness activities, including a national stakeholder meeting on the MISP, training on long-acting reversible contraceptives (LARC), orientation to Sexual and Gender Based Violence in Emergencies (SGBViE), and attendance at cluster meetings and interagency coordination with stakeholders.

In 2017, IPPF Humanitarian Pacific team members and the TFHA hosted a national stakeholder meeting to orient participants on SRHiE and the MISP. This meeting was followed by a more detailed MISP training conducted by the IPPF Humanitarian Pacific Hub in partnership with TFHA and supported by the SPRINT program. Twenty-four volunteers/ first responders participated in this MISP training in the capital, Nukualofa. In addition to MISP orientation and training sessions, TFHA ran LARC training to incorporate this into future service provision and an orientation to SGBV. Training was delivered alongside regular preparedness activities and participation in national stakeholder meetings with the National Emergency Management Office, MoH, Ministry of Internal Affairs, Emergency Services (including Police), and local NGOs working in women’s rights, disability, and lesbian, gay, bisexual, transgender, queer, intersex (LGBTQI) areas.

Training also continued during the response when gaps in the provision of psychosocial support for SGBV survivors were identified, especially on the island of ‘Eua. A half-day orientation on SGBV in emergencies was conducted in 2018 for field responders, facilitated by UNFPA and supported by SPRINT response funding in collaboration with TFHA, IPPF Pacific Humanitarian Hub, and the MoH. A total of 42 Tongatapu-based clinical staff nurses and midwives were trained in basic concepts and fundamental guiding principles in dealing with a range of SGBV issues. Gita, therefore, provided the opportunity to upskill clinical staff, building competence, networks, and relationships.

Key informants also noted that the TFHA staff had attended several cluster meetings as a key stakeholder. These included meetings with the Health, Nutrition, Water, Sanitation and Hygiene (HNWASH) cluster and the Safety and Protection cluster involving the MoH, UN agencies and NGOs.

### Responding to sexual and reproductive health needs after cyclones Winston and Gita

An SRH response was launched in the aftermath of both Tropical Cyclones Winston and Gita. The scope of these responses differed, and Table 1 summarises these against the objectives of the MISP (2010). Key differences are seen in preventing and responding to sexual violence and planning for comprehensive SRH services, integrated into primary care. Safe and rational blood transfusion in place was not reported in either setting.

**Table 1:** Summary of activities and gaps in the SRHiE response to Cyclones Winston (Fiji) and Gita (Tonga)

MISP (2010) objective and activities	Fiji response: Winston	Tonga response: Gita
Objective 1: Ensure health cluster/sector identifies agency to LEAD implementation of the MISP		
Activity 1: RH Officer in place	√	√
Activity 2: Meetings to discuss RH implementation held	√	√
Activity 3: RH Officer reports back to the health cluster/ sector	√	√
Activity 4: RH kits and supplies available and used	√	√
Objective 2: Prevent Sexual Violence and assist survivors		
Activity 1: Protection system in place especially for women and girls	Somewhat: monitoring is undertaken; information sessions conducted	√
Activity 2: Medical services and psychosocial support available for survivors	Some services and referral available	√
Activity 3: Community aware of services	√	√
Objective 3: Reduce the transmission of HIV		
Activity 1: Safe and rationale blood transfusion in place	–	–
Activity 2: Standard precautions practices	During medical missions	During medical missions
Activity 3: Free condoms available	√	√
Objective 4: Prevent excess maternal and neonatal mortality and morbidity		
Activity 1: EmONC services available	√	√
Activity 2: 24/7 referral system established	Referral (at time of medical mission)	√
Activity 3: Clean delivery kits provided to birth attendants and visibly pregnant women	√	√
Activity 4: Community aware of services	√	√
Objective 5: Plan for comprehensive RH services, integrated into primary health care		
Activity 1: Background data collected	√	√
Activity 2: Sites identified for future delivery of comprehensive RH	–	√
Activity 3: Staff capacity assessed and trainings planned	–	√
Activity 4: RH equipment and supplies ordered	–	–
Additional Priorities:		
Continue family planning	√ and new users	√ and new users
Manage symptoms of STIs	√	√
Continue HIV care and treatment	–	√
Distribute hygiene kits and menstrual protection materials	√	√

*EmOC* emergency obstetric care, *RH* reproductive health

#### Fiji

The training at the onset of the cyclone response led trainees, with the support of surge staff, to initiate a Family Health Sub-cluster to facilitate a collaborative SRH response with the MoH, medical services teams and partners. According to one key informant, this was essential “otherwise reproductive health would have been lost in the health cluster because they had so many other concerns”.

Before the guidance that was provided during this training, staff had found this a challenging time.

we had to learn which cluster meetings to go to. We had to see where we fit into the security one and the health clusters. Even in the health clusters, we had to fight even to have a reproductive health cluster within the health cluster which wasn’t there before… That’s why we were so disadvantaged, there was a lot to handle.

Links with the MoH also required strengthening. One informant said:

there was collaboration, there was an existing memorandum of understanding with the Ministry of Health but it dipped a bit and that relationship sort of was estranged… During TC Winston, there would have been, there was a relationship, but it wasn’t an active, engaged relationship shall we say during TC Winston.

It was therefore reported that “we had to make extra efforts to be brought in”. These ‘extra efforts’ in the form of advocacy by motivated IPPF SROP and MA representatives and guided by surge capacity staff led to the establishment of the sub-cluster in collaboration with Ministry of Health and Medical Services, and the delegation of responsibility to RFHAF- achievements regarded as impressive by several respondents. They also strengthened the relationship with government, an outcome explained by one respondent as:

crucial because these are the things that will really hinder you, will make it very difficult for one humanitarian team to operate if you do not have the support from your own leadership and if the government doesn’t trust you.

While informants noted initial uncertainty regarding which cluster meetings to attend, they were also aware of general confusion at the time of the response “at that time… there were so many organisations that came in with different agendas and they wanted to be the first in.” Despite this, all agreed that coordination had improved post-Winston and support had increased since the establishment of the IPPF Humanitarian Pacific Hub, with one respondent stating that the situation is:

[better] coordinated, not like before when we were looking and finding ways with the existing system of the government, but now we know after the MISP, after the set-up of the humanitarian arm here, it’s more coordinated and it’s quicker.

Medical missions were launched the day after the brief training in Fiji. The RFHAF/IPPF SPRINT team delivered family planning counselling and referred pregnant women in their third trimester to birthing units. They distributed clean delivery kits, contraceptives, and dignity kits (containing sarongs, undergarments, thongs, whistles, soap, and sanitary pads).

The team also provided safe spaces for displaced women and girls and community awareness on GBV, though skill weaknesses in this area were noted:

we were just at that point strengthening the objective two components of MISP and so I think at that time we couldn’t even consider ourselves a player at that point because we were not involved in the GBV or the protection work in Fiji.

Lessons were learnt and applied as the SRH response progressed, with one responder reflecting that:

the first intervention… was really disorganised, but after that when we came to the second one we were able to take a lot of lessons and even recommendations from the community about how we could do it best and we even incorporated that intervention when we went to the west.

The collation of supplies and logistics also delayed the medical response as no action had been taken for securing these during the preparedness phase. One informant said: “what delayed our trip was we had to buy the stuff and get our dignity kits.” Another stated:

at that time we were trying to rent vehicles and they were all out… And that was a drawback because we were a bit late in our response… There was no coordination and we should have booked the car but we had all these competing agendas.

Roles were not always clear to responders who reported taking on many functions:

So, I was everywhere. I don’t really understand what was my role at that time because I seemed to be doing everything! I coordinated, I went to the village headmen, I went to the Ministry of Health for meetings, then I wore my nursing cap when I gave the injection and I was also the driver.

In addition to MISP work, staff were engaged in activities that were not related to SRH:

The Chief of the village we visited was sick. And because it was so far away up the mountains and there was no transport, we had to get the Chief man, because he had something that needed medical attention and because we were there, we had to drive him down to the main hospital. But we had to do it. And after a hurricane it’s not that easy to drive the Fiji roads where you have bridges washed away and big potholes. So that was something besides the MISP that we did during our response.

However, informants stated that such activities were necessary to build rapport, and the willingness of staff to accommodate these additional needs was well-regarded by recipient communities. Some challenged the importance of SRH response, believing that the focus should be shelter and food. This required a strategic and respectful approach:

It’s actually about convincing the masses why it is important. It was not an easy job but we were able to tell them, during a disaster and after… women won’t stop having babies during a disaster…The communities came to appreciate that and that was quite a good feeling.

IPPF surge staff remained with the in-country and SROP-supported response teams in Fiji for 10 days to advise, guide, and debrief. One informant recalled:

The good thing about it is after every village we went to, no matter how late it was we would sit together as a team… and go through the day… We built on our lessons learnt every day and we had [the two support persons] there and they were really observers when we provided the service except the doctors and counselling. But they would attend the information sessions and go in and see how we would demarcate the areas and the signs and they would help explain properly and they would feedback to us in the evening.

More broadly, it was reported that knowledge, skills and relationships developed during this response have been utilised and built upon in subsequent preparedness efforts and humanitarian action. Further advocacy for the integration of SRHiE in emergency preparedness plans; collaboration with government at various levels for capacity development; training of clinical, program and volunteer staff in-country; and coordination with other key NGOs have been undertaken by RFHAF and supported by the IPPF Humanitarian Pacific Hub, established since Winston, “all geared towards being MISP ready and having strong systems in place” (Respondent).

#### Tonga

Key informants were optimistic about the response to Gita, explaining that “overall, the response was good and the TFHA team felt they were in control”. Staff were described as highly motivated, with one informant declaring: “it was new for us and became very exciting for us to provide the MISP, and we were able to get DFAT, who is the donor, to join us on one of our visits and they were happy with what we showed”. Comparisons were made with the response in Fiji and one key informant stated:

Tonga [the population] is much smaller [than Fiji] and the [TFHA] members as well have a very strong relationship with the Ministry of Health. I think those two things, there were a few things to their advantage. For example, one of the National Disaster Management Office coordinators actually sits on the Tonga Family Health board and also a Ministry of Health officer.

In addition, relationships and networks developed with the MoH, NGOs and communities during preparedness activities were easily activated in response to Gita. When the MoH made an official request to the TFHA to facilitate SRH services and education to communities affected by Tropical Cyclone Gita on 19th February 2018, the TFHA formed a “core team” with the MoH and NGOs to undertake these activities in coordination with the HNWASH and Safety and Protection Clusters.

One individual stated that the TFHA had “a very good relationship with the Australian DFAT (Department of Foreign Affairs and Trade) post in Tonga, maybe because they’re just down the road. There’s that active engagement even during normal times”. However, there was still “a rapid learning curve” when it came to moving from the training room to disaster implementation. The assistance provided by the IPPF Humanitarian Hub including the training and development of a response plan and proposal for funding was regarded as a “big advantage “by a key informant.

Staff roles were expanded when the TFHA team agreed with the MoH to include cervical cancer, diabetes and high blood pressure screening in the response “given the high burden of non-communicable disease in the Tongan community “. As in Fiji, it was identified that staff lacked capacity to address objective 2 of the MISP, responding to sexual violence. They instituted a brief training intervention to increase the capability of nurses to counsel and refer identified cases.

Despite informants expressing satisfaction with the response, some pointed to necessary improvements including the need to better think through transport to outer islands as staff had to rely on fishing boats, and tailoring dignity kits to suit the local context. Plans to improve preparedness were in train including undertaking MISP readiness assessment, the integration of the MISP into the national reproductive health policy, and lobbying to include the MISP in the Tongan Government’s goal to respond within the first 72-h of an emergency.

#### Shared insights

Key informants agreed on several issues, including that preparation is key for any response and that this must include hands on skill development and building and maintaining strategic relationships and community links. As explained by one participant:

80% of your response lies in how prepared you are. And being prepared doesn’t just mean that you have clinicians trained, or the resources prepositioned, it’s about being part of a national support network… we need to have those linkages to national level. We need to have those policies in place, we need to have the buy in from the key ministries…and I think we need to have partnerships- these play a great deal in the preparedness needs. And definitely capacity building at the MA level not just for the clinical or program staff but for youths engaged, at the board level for governance and so people are clear about what their role is and how that contributes to the bigger, broader picture of meeting people’s SRH needs.

The engagement and motivation of SPRINT-supported individuals and teams was regarded as an important driver of the response in both settings. This was seen in the many efforts to overcome obstacles in Fiji and Tonga nd the commitment to dedicate long hours and “heavy work” (Respondent) to meeting the needs of affected communities. This, combined with technical knowledge developed through capacity development was described as key:

You need passion and technique. For humanitarian response, you can teach technique, but you can’t teach motivation and passion…That’s why I was confident with any response, as long as I’m working with the right people. And these were the right people on the ground... But they need the knowledge and that knowledge, that technique, can be provided through training and support.

Respondents from both Tonga and Fiji noted a lack of systemic data collection on the status of vulnerable and marginalised groups during the response. This lack of data was seen as a barrier to mobilising an effective SRHiE response and planning future responses. In Tonga, this need for reliable data was reported to extend beyond particular groups to a general shortage of demographic and health-related data at a country level. One informant called for “standards for reporting and country appropriate indicators to allow the comparison of responses.” While UNFPA provided commodities for distribution, they did not assume an implementing function during the cyclone responses. It was suggested, however, that UNFPA involvement in monitoring and evaluation would have benefited the response in both countries.

## Discussion

This study found that differences in Fiji and Tonga’s preparedness, at the individual, organisational and systems levels prior to Tropical Cyclones Winston and Gita, influenced the type, scope, and timeliness of the sexual and reproductive health response. In Fiji, activities were concentrated on IPPF support to provide training to rapidly scale-up the capacity of responders at the onset of the disaster, and to strengthen relationships and access to platforms for coordination. In Tonga, individual and organisational capacity had already been established alongside inter-organisational networks across the sector and at the national level. Respondents in Tonga reported feeling prepared and confident. This is likely to be linked to the investment in preparedness activities and capacity building before Gita that was not present in Fiji before Winston. Despite an existing memorandum of understanding, with the Fiji MoH, regular communication appears to have lapsed. In contrast, considerable work had been undertaken in Tonga to build and maintain relationships with the government, NGOs and communities for SRH response. These capacity-building and preparedness activities in Tonga allowed the response team to take clear and directed action, engage with established coordination partners and platforms, and implement a relatively harmonised response. The gaps in preparedness in Fiji meant that there was a lack of clarity in initial efforts, and time was lost at the onset of the response. Despite these early challenges, however, adaptations were made to capitalise on the motivation, existing capabilities, position, and relationships of those involved in the response to Winston.

This study found that a range of approaches to staff capacity building, such as regular in-service workshops in Tonga and rapid training at the onset and during the disasters in both countries, followed by mentoring and support, motivated and engaged staff in the provision of SRH and broader health services to affected communities. This emphasises the need for regular, on-going training and supportive strategies that are relevant and contextualised. Training is often the focus of capacity development [28], and a review of organisational change in the sector [29] concluded that training is only weakly linked to actual practice in humanitarian agencies and therefore needs to be supported by other capacity development initiatives. The limited effectiveness of training programs highlights the need for training to be situated within a set of buttressing strategies so that staff can apply knowledge and skills in the field. Pearson states that the design of training interventions “should be informed by an in-depth understanding of the context and the identification of opportunities and constraints, and appropriately aligned to broader [capacity development] initiatives” (2011 p9). A systematic review of studies examining the transfer of training into practice for SRH in humanitarian settings found that individual, training, organisational, socio-cultural, political and health system factors all contribute to the ability of trainees to apply newly acquired knowledge and skills in their work settings [30]. This highlights the need for comprehensive activities at multiple levels within a country and across the Pacific region to build capacity for an SRH response.

The training and subsequent mentoring and technical support provided by IPPF surge staff was reported to be indispensable in Fiji, highlighting the importance of these buttressing strategies to support capacity development efforts and optimise the application of knowledge and skills to action. Our study found that informants highlighted the importance of learning by doing, of feedback and support, and of building capacity through the process of implementation across both country contexts. Role flexibility was noted along with the need to be adaptable in incorporating non-SRH response activities as relevant to the local context.

Factors at an organisational level also influenced the SRH response in both contexts. The support of management and program staff and the availability of surge capacity and technical guidance was widely appreciated. While this, together with the formalisation of partnerships and regular meetings and training are important activities to ensure the currency of coordination efforts in readiness for activation, so is the institutionalisation of SRHiE in national policy and accountability mechanisms [31]. National policies that highlight SRHiE as a priority and embed the MISP into disaster risk reduction (DRR) planning with attached investment and key performance indicators support the delivery of essential services in emergencies. The latest Fijian National Disaster Risk Reduction policy 2018–2030, post-cyclone Winston, notes the challenges of gender-based violence and that reproductive health services are likely to be disrupted during disasters. While it includes strategies to support specific groups such as pregnant women and LGBTQI people, the policy stops short of noting the MISP [32].

It has been noted that while the SPRINT initiative has been implemented across several regions to improve organisational and national capacity, preparedness training for communities across the sector more broadly has been largely neglected [33]. At the same time, our research notes that both RFHAF and TFHA have established relationships with communities, and that they could be further supported to prepare for and better respond to disasters. One approach to building relationships could be through participatory training activities with communities using available curriculum in reproductive health and gender [34]. While preparing for anticipated disaster scenarios through training is important so is the ability of individuals, organisations and communities to adapt and be flexible to apply skills to new situations and problems.

The COVID-19 pandemic has provided an opportunity to examine more localised ways to address the provision of SRHR and build national and regional capacity to improve disaster risk reduction strategies and plans. A Western Pacific Regional Action Plan for Response to Large-Scale Community Outbreaks of COVID-19 has been developed [35]; however, SRHR is notably absent in this document. Despite this, Pacific Island nations, including Fiji and Tonga have implemented various strategies to ensure a SRHR response, demonstrating their resilience and innovation [36]. Much work remains to be done to better build and connect capacity strengthening activities from the individual to national levels, not just for preparedness and response but for recovery and mitigation efforts.

### Limitations

This study is limited by the small number of participants; however, those interviewed were key informants involved in the decision making during the preparedness and response phases of cyclones Winston and Gita. Insights from a diversity of participants in Fiji and Tonga may have provided further detail regarding the activities that were undertaken. These interviews were conducted some- time after the cyclones and the memories of some informants may have been compromised; however, this study was focused on high-level activities, and many had prepared for the interviews by consulting internal documents. Multiple contacts with participants enabled the researchers to follow up on details and check information with informants. We were mindful of possible social desirability bias and interview data was assessed for both positive and negative responses and imbalances were not noted.

## Conclusion

This research has outlined the need for comprehensive activities at multiple levels within a country and across the Pacific region to build capacity for an SRH response in crisis situations. While the SPRINT initiative has been implemented across several regions to improve organisational and national capacity preparedness activities, training for communities can be strengthened. The study highlights the importance of formal partnerships, regular communication, institutionalising SRH in policy and accountability mechanisms, and training to ensure coordination efforts are up-to-date in disaster readiness.

## Data Availability

De-identified data is available upon request.

## Abbreviations

GBV: Gender-based violence
IPPF: International planned parenthood federation
LARC: Long-acting reversible contraceptives
LGBTQI: Lesbian, gay, bisexual, transgender, queer, intersex
MISP: Minimum initial service package for sexual and reproductive health in crisis situations
MoH: Ministry of health
NGO: Non-Government Organisation
OHCHR: Office of the United Nations High Commissioner for Human Rights
RFHAF: Reproductive and Family Health Association of Fiji
SROP: Sub-regional office
SRHiE: Sexual and reproductive health in emergency
SRHR: Sexual and reproductive health and rights
SPRINT: Sexual and reproductive health programme in Humanitarian settings
STIs: Sexually transmitted infections
TFHA: Tonga Family Health Association
UN: United Nations
WHO: World Health Organization
